# Supplementary material for: P-Glycoprotein (ABCB1/MDR1) and BCRP (ABCG2) Limit Brain Accumulation and Cytochrome P450-3A (CYP3A) Restricts Oral Exposure of the RET Inhibitor Selpercatinib (RETEVMO)
Source: Pharmaceuticals (Basel). 2021 Oct 27;14(11):1087. doi: 10.3390/ph14111087 (PMC8617681; doi:10.3390/ph14111087)
Supplement: Supplementary file 1 [file pharmaceuticals-14-01087-s001.zip › pharmaceuticals-1403966-supplementary.pdf]

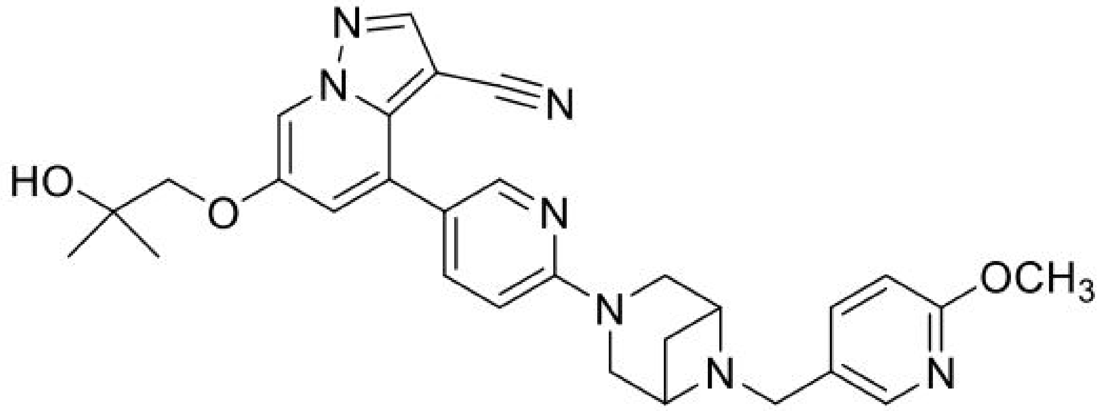

Supplemental Figure S1. Molecular structure of selpercatinib (RETEVMO, LOXO-292, 525.6 g/mol).

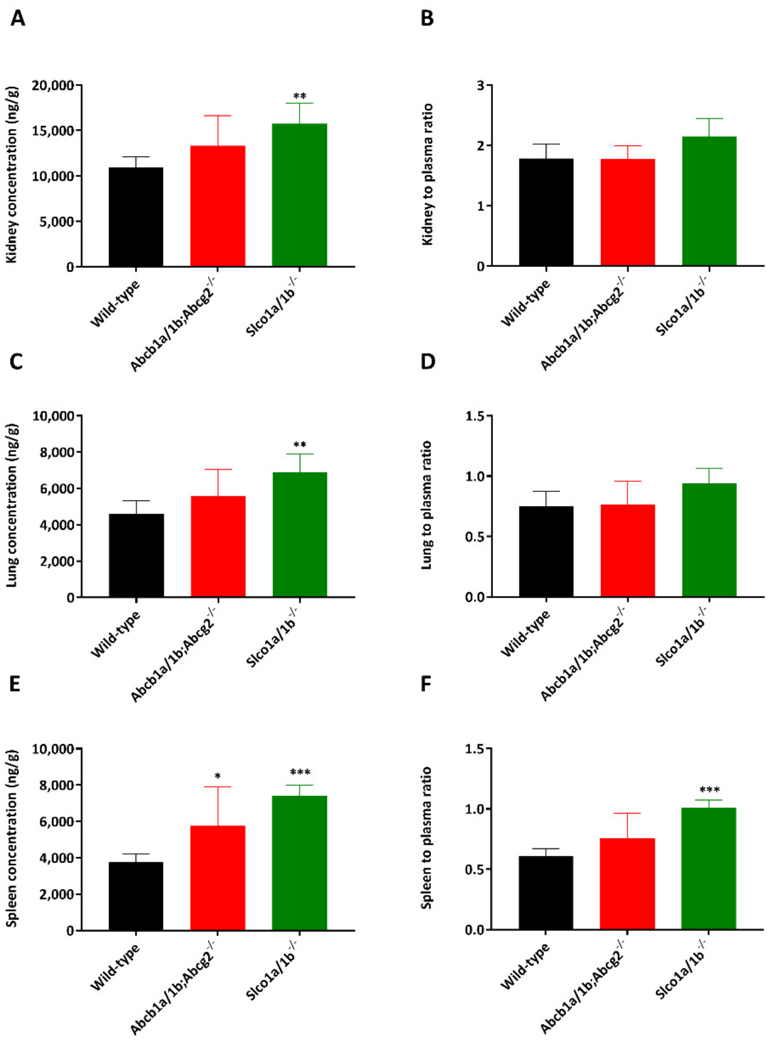

Supplemental Figure S2. Tissue concentrations (A, C, E) and tissue-to-plasma ratios (B, D, F) of selpercatinib in male wild-type, *Abcb1a/1b;Abcg2*$^{-/-}$ and *Slco1a/1b*$^{-/-}$ mice 4 h after oral administration of 10 mg/kg selpercatinib (n = 6 - 7). *, $P < 0.05$; **, $P < 0.01$; ***, $P < 0.001$ compared to wild-type mice. Statistical analysis was applied after log-transformation of linear data.

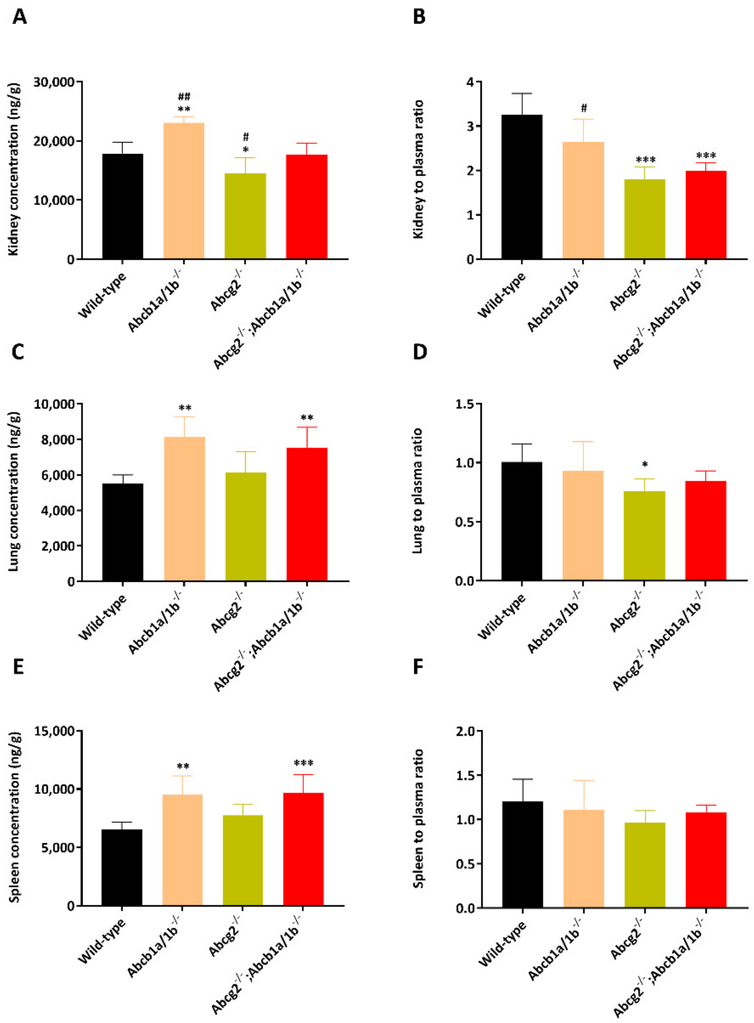

Supplemental Figure S3. Tissue concentrations (A, C, E) and tissue-to-plasma ratios (B, D, F) of selpercatinib in male wild-type, *Abcb1a/1b*$^{-/-}$, *Abcg2*$^{-/-}$ and *Abcb1a/1b;Abcg2*$^{-/-}$ mice 4 h after oral administration of 10 mg/kg selpercatinib (n = 6). *, $P < 0.05$; **, $P < 0.01$; ***, $P < 0.001$ compared to wild-type mice; #, $P < 0.05$; ##, $P < 0.01$; ###, $P < 0.001$ compared to *Abcb1a/1b;Abcg2*$^{-/-}$ mice. Statistical analysis was applied after log-transformation of linear data.

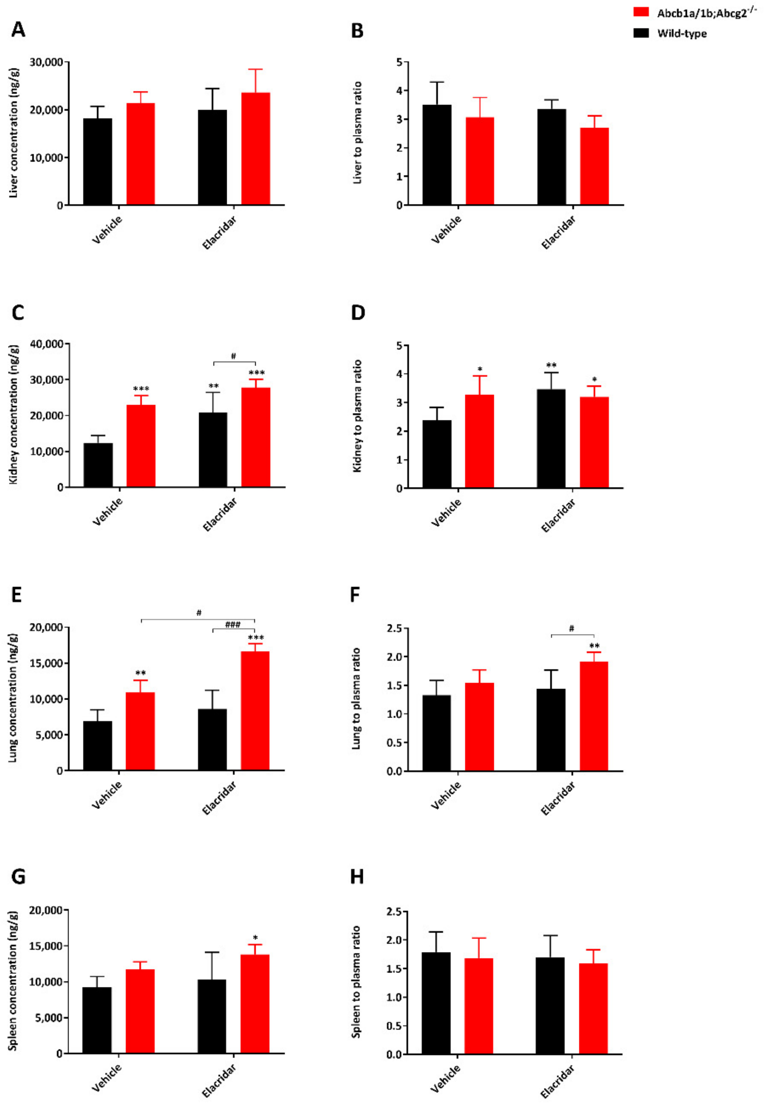

Supplemental Figure S4. Tissue concentrations (A, C, E, G) and tissue-to-plasma ratios (B, D, F, H) of selpercatinib in male wild-type and *Abcb1a/1b;Abcg2*$^{-/-}$ mice over 2 h after oral administration of 10 mg/kg selpercatinib with or without co-administration of elacridar. Data are given as mean ± S.D. (n = 6). *, *P* < 0.05; **, *P* < 0.01; ***, *P* < 0.001 compared to wild-type mice; #, *P* < 0.05; ##, *P* < 0.01; ###, *P* < 0.001 compared among other groups. Statistical analysis was applied after log-transformation of linear data.

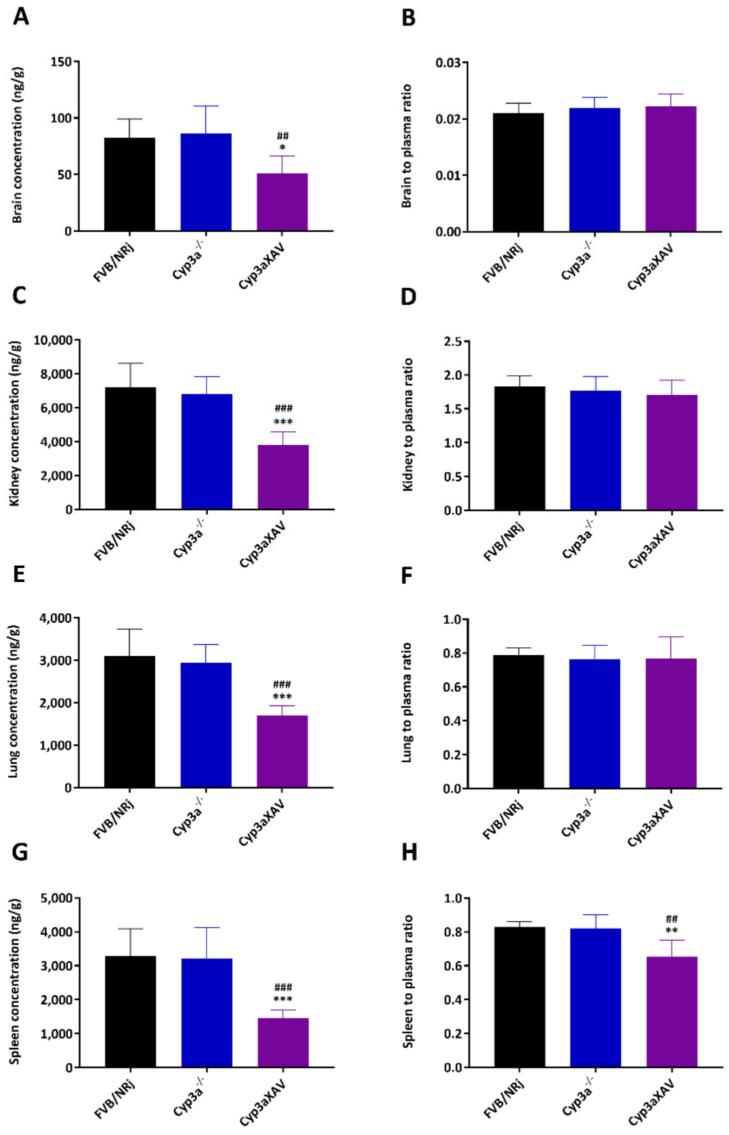

Supplemental Figure S5. Tissue concentrations (A, C, E, G) and tissue-to-plasma ratios (B, D, F, H) of selpercatinib in female wild-type, *Cyp3a*$^{-/-}$ and Cyp3aXAV mice over 8 h after oral administration of 10 mg/kg selpercatinib. Data are given as mean ± S.D. (n = 6 - 7). *, *P* < 0.05; **, *P* < 0.01;***, *P* < 0.001 compared to wild-type mice; #, *P* < 0.05; ##, *P* < 0.01; ###, *P* < 0.001 compared between *Cyp3a*$^{-/-}$ and Cyp3aXAV mice. Statistical analysis was applied after log-transformation of linear data.

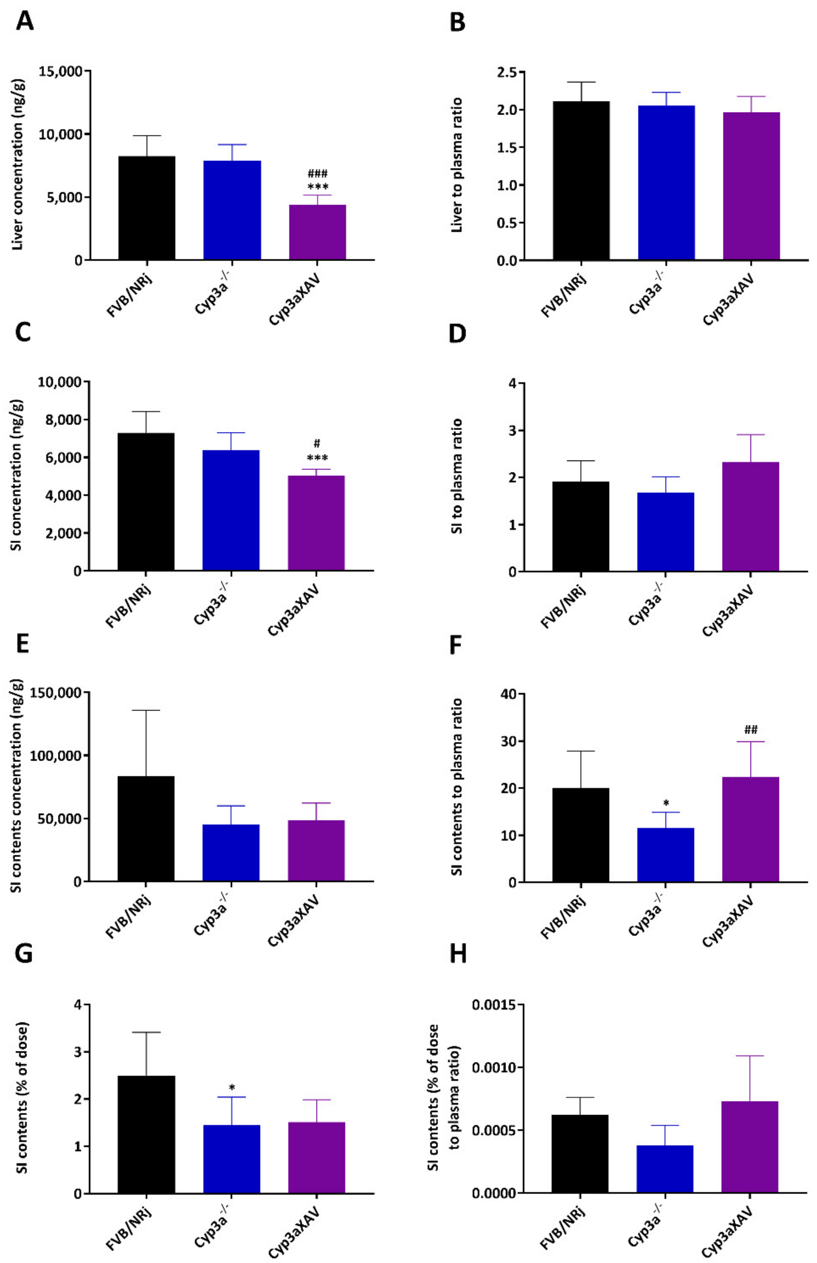

Supplemental Figure S6. Liver, small intestine and small intestine contents concentrations (A, C, E), liver, small intestine- and small intestine contents-to-plasma ratios (B, D, F), small intestine contents as percentage of dose (G) and small intestine contents percentage of dose-to-plasma ratio (H) of selpercatinib in female wild-type, *Cyp3a$^{-/-}$* and Cyp3aXAV mice over 8 h after oral administration of 10 mg/kg selpercatinib. Data are given as mean ± S.D. (n = 6 - 7). *, $P < 0.05$; **, $P < 0.01$; ***, $P < 0.001$ compared to wild-type mice; #, $P < 0.05$; ##, $P < 0.01$; ###, $P < 0.001$ compared between *Cyp3a$^{-/-}$* and Cyp3aXAV mice. Statistical analysis was applied after log-transformation of linear data.

Supplemental Table S1. Pharmacokinetic parameters of selpercatinib in male wild-type, *Abcb1a/1b;Abcg2*$^{-/-}$ and *Slco1a/1b*$^{-/-}$ mice over 4 h after oral administration of 10 mg/kg selpercatinib.

Parameter	Genotype		
	Wild-type	*Abcb1a/1b;Abcg2*$^{-/-}$	*Slco1a/1b*$^{-/-}$
AUC_{0-4h}, ng/ml*h	26,649 ± 6,360	30,188 ± 7,632	36,197 ± 5,255
Fold change AUC_{0-4h}	1.0	1.1	1.4
C_{max}, ng/ml	7,862 ± 1,814	8,582 ± 2,160	11,625 ± 1,614*
T_{max}, h	1.8 ± 1.2	1.6 ± 1.2	1.7 ± 0.52
C_{brain}, ng/g	186 ± 23	3,454 ± 855***	278 ± 42**
Fold increase C_{brain}	1.0	18.6	1.5
Brain-to-plasma ratio	0.030 ± 0.004	0.46 ± 0.04***	0.038 ± 0.005**
Fold increase ratio	1.0	15.3	1.3
C_{liver}, ng/g	17,593 ± 3,471	19,077 ± 2,696	19,916 ± 3,174
Fold increase C_{liver}	1.0	1.1	1.1
Liver-to-plasma ratio	2.8 ± 0.4	2.6 ± 0.3	2.7 ± 0.4
Fold increase ratio	1.0	0.93	1.0
C_{SIC}, ng/g	91,051 ± 22,029	34,929 ± 16,659*	140,998 ± 48,076
Fold change C_{SIC}	1.0	0.38	1.5
SIC-to-plasma ratio	14.9 ± 4.1	4.5 ± 1.8***	19.0 ± 5.3
Fold increase ratio	1.0	0.30	1.3
C_{testis}, ng/g	730 ± 103	5,726 ± 1,535***	1,121 ± 107**
Fold increase C_{testis}	1.0	7.8	1.5
Testis-to-plasma ratio	0.12 ± 0.02	0.76 ± 0.13***	0.15 ± 0.01*
Fold increase ratio	1.0	6.3	1.3

AUC_{0-4h}, area under plasma concentration-time curve; C_{max}, maximum concentration in plasma; T_{max}, time point (h) of maximum plasma concentration; C_{brain}, brain concentration; C_{liver}, liver concentration; SIC, small intestine contents; C_{SIC}, small intestine contents concentration; C_{testis}, testis concentration;. Data are given as mean ± S.D. (n = 6 - 7). *, $P < 0.05$; **, $P < 0.01$; ***, $P < 0.001$ compared to wild-type mice. Statistical analysis was applied after log-transformation of linear data.
